# Blepharospasm, Oromandibular Dystonia, and Meige Syndrome: Clinical and Genetic Update

**DOI:** 10.3389/fneur.2021.630221

**Published:** 2021-03-29

**Authors:** Hongying Ma, Jian Qu, Liangjun Ye, Yi Shu, Qiang Qu

**Affiliations:** ^1^Department of Pharmacy, Xiangya Hospital, Central South University, Changsha, China; ^2^National Clinical Research Center for Geriatric Disorders, Institute for Rational and Safe Medication Practices, Xiangya Hospital, Central South University, Changsha, China; ^3^Department of Pharmacy, The Second Xiangya Hospital, Central South University, Changsha, China; ^4^Institute of Clinical Pharmacy, Central South University, Changsha, China; ^5^Department of Pharmacy, Hunan Provincial Corps Hospital of Chinese People's Armed Police Force, Changsha, China; ^6^Department of Neurology, The Second Xiangya Hospital, Central South University, Changsha, China

**Keywords:** Meige syndrome, blepharospasm, oromandibular dystonia, pathogenic gene, variants

## Abstract

Meige syndrome (MS) is cranial dystonia characterized by the combination of upper and lower cranial involvement and including binocular eyelid spasms (blepharospasm; BSP) and involuntary movements of the jaw muscles (oromandibular dystonia; OMD). The etiology and pathogenesis of this disorder of the extrapyramidal system are not well-understood. Neurologic and ophthalmic examinations often reveal no abnormalities, making diagnosis difficult and often resulting in misdiagnosis. A small proportion of patients have a family history of the disease, but to date no causative genes have been identified to date and no cure is available, although botulinum toxin A therapy effectively mitigates the symptoms and deep brain stimulation is gaining increasing attention as a viable alternative treatment option. Here we review the history and progress of research on MS, BSP, and OMD, as well as the etiology, pathology, diagnosis, and treatment.

## Introduction

Blepharospasm (BSP), oromandibular dystonia (OMD), and Meige syndrome (MS) are different movement disorders that are different but closely related. MS is cranial dystonia characterized by the combination of upper and lower cranial involvement and includes BSP and involuntary movements of the OMD. Most researchers and clinicians would agree that MS, BSP, and OMD are not one single entity, but rather a clinical syndrome of multifactorial origin. Atypical parkinsonism patients frequently have the BSP symptom, and some patients with Huntington's disease have OMD. The causes of BSP, OMD, and MS remain elusive, but some convincing evidence suggests that these disorders are a multifactorial disease, where one or more unknown genes, as well as epigenetic and environmental factors, are combined to reach the disease threshold (1). Previously, according to etiology, dystonia has been classified as primary (dystonia is the only clinical sign and includes idiopathic or genetic disorders with no neuropathological abnormalities) or secondary [dystonia arising from neurodegeneration, acquired causes (such as lesions within the brain), or genetic conditions with a progressive course] (2). The 2013 consensus update on the phenomenology and classification of dystonia focuses on clinical characteristics and classifies dystonia as isolated (dystonia is the sole manifesting clinical feature with no other neurological or systemic signs) or combined (dystonia is combined with other neurological or systemic signs) (3). In this review, we provide a comprehensive overview of the new findings regarding the isolated/primary dystonias of BSP, OMD, and MS. in the past decades and highlight what gaps remain in our knowledge of this condition.

## Relationship Between BSP, OMD, and MS

BSP is a primary adult-onset focal dystonia characterized by involuntary closure of the eyelids and spasms of the orbicularis oculi muscles (4). Over time, some patients with BSP develop OMD, which involves involuntary lower facial and masticatory movements including lip pursing, chewing, and jaw opening/clenching (5). MS is named for the French neurologist Henri Meige, who originally described a combination of BSP and involuntary movements of the lower facial and/or masticatory (jaw) muscles (6). In 1976, the British neurologist David Marsden diagnosed a case of BSP combined with oral and mandibular dystonia as Brueghel syndrome (7).

According to clinical characteristics of dystonia, dystonia is classified by age at onset, body distribution, temporal pattern, the coexistence of other movement disorders, and other neurological manifestations (3). Body distribution of dystonia comprises focal, segmental, generalized (with or without leg involvement), multifocal, and hemidystonia (3). Focal dystonia involves only one part of the body and encompasses BSP, spasmodic dysphonia, and handwriting spasm. Segmental dystonia involves two or more contiguous body regions and typical examples of segmental forms are cranial dystonia (BPS with lower facial and jaw or tongue involvement) or bibrachial dystonia (3, 8). MS is an example of the cranial subtype that manifests as a combination of BSP and OMD (9); the disorder may initially be limited to BSP or OMD only, and then later spread to other muscles (9).

Etiology classification of dystonia compromises two complementary characteristics that may be useful for classification: identifiable anatomical changes and pattern of inheritance. The term “primary” is currently used as an etiological descriptor for genetic or idiopathic cases where dystonia is isolated and there is no consistent pathologic change (3). This dual meaning does not help clarity and the use of the term primary is currently discouraged (3). Inherited dystonia forms have proven genetic origin; acquired dystonia due to a known specific cause; idiopathic dystonia has unknown causes. Therefore, MS, BSP, and OMD could be also classified into idiopathic, acquired, and inherited subtypes.

With our in-depth study of disease pathology, clinical diagnostic guidelines are constantly updated. To date, specific diagnostic guidelines of adult-onset focal dystonia have been proposed and validated for BSP and laryngeal dystonia (10, 11). Expert recommendations for diagnosing cervical, OMD, and limb dystonia were also published (12). Moreover, new clinical evaluation tools were invented such as a soft, flexible hybrid bioelectronic system that offers highly conformal, gentle lamination on the skin, while enabling wireless, quantitative detection of electrophysiological signals (13). The wearable bioelectronics outperforms the conventional manual clinical rating for BSP patients (13).

## Clinical Manifestations and Epidemiology

The clinical features of MS vary widely among patients, which have different phenotypic forms, ranging from tonic spasm or prolonged closure of the eye, clonus of orbicularis oculi to complete inability to open the eyes as eyelid weakness or blepharoptosis is also very common (14). It may appear first as unilateral BSP that will later become bilateral (14). As the disease progresses, the involvement of the lower facial and masticatory muscles becomes fairly common in patients with BSP, including lip pursing, chewing, jaw thrusting, grimacing, jaw opening, and jaw closing/clenching (5). As the disease progresses, extensive multiple muscle groups show hyperactivity, with a full disease state observed after 1–4 years (15). Spasms normally last for several to tens of seconds before expanding to other areas. Dystonia can persist for several minutes while spasms become less synchronous (16–18). Clonic contraction or hyperactivity can also precede tonic contraction (16–18). Besides, some reports were showing that in elderly female patients with a history of head injury and BSP, dystonia can more easily spread to other parts of the body (5, 19, 20). The probability of spread to contiguous muscles in BSP is a very common (>50%) phenomenon. It happened highest during the initial 3–5 years of BSP onset, with nearly half of the spread occurring during the first year of illness, with rare instances recorded when the spread was delayed for a decade or more (15, 21). About 50% of patients with BSP develop dystonia in other areas, and some patients experience dry eyes and rigid pupils (22). In 155 BSP patients study by Defazio et al., previous trauma to head or face with unconsciousness, age at onset of BSP, and female sex were the predictors of the spread (23). Berman et al. analyzed 487 adult-onset isolated focal dystonia including 50% of BSP and found that the most common regions for first spread were the oromandibular region (42.2%) and neck (22.4%) for BSP (24). Increased spread risk was associated with positive family history and self-reported alcohol responsiveness (24).

Sensory tricks are common among patients with MS, which are the sensory stimuli, learned by the patients to alleviate the dystonia. Sleeping, relaxing, talking, pulling the upper eyelid, blowing cheeks, walking, exposure to cold water, yawning, or drinking beverages can all alleviate dystonia. More than half of the patients with BSP have one or more sensory tricks (14). Percham et al. reported sensory trick in 87% of BSP patients including MS patients and the majority had more than one sensory trick (15). The most common tricks were touching above the eyes, singing, humming, and talking (9). Spontaneous remissions in BSP and MS are rare and seen in <10% of the BSP and MS patients and usually tend to occur in the first 5 years of symptom onset (25, 26).

The prevalence was a range of 13–130 cases per million for BSP and 69 per million for OMD in the US (27). The prevalence of BSP accounts for 36 per million (95% CI 31–41) in Europe in 2000 (28). BSP and MS are significantly more common in females than males and the male: female ratio is 1:2 (9). The average age of MS onset is in the sixth decade and age appears to be an independent risk factor in the development of MS (9). The average age of onset for BSP is around 55 years whereas the average age of onset for OMD is only a couple of years earlier (5). Women are at more risk than men because of specific estrogen receptors influencing involuntary motor function (9, 14). In some cases, there is a family history of the disease (29–31), and MS or other forms of dystonia are present in up to 10% of first- or second-degree relatives of patients (32–35).

## Pathophysiology

The pathophysiology of MS is not clear enough. A line of evidence suggests that the pathophysiology of dystonia involves the striatum, whose activity is modulated among other neurotransmitters, by the dopaminergic system (36). The most widely accepted hypothesis for the pathogenesis of MS is dopaminergic and cholinergic abnormalities (14, 36). Recent studies using neurophysiological and neuroimaging techniques have supported that environmental triggers and genetic predisposition cause plastic changes and reduced cortical inhibition (9). A voxel-based morphology analysis investigated the changes and clinical significance of brain structural abnormalities in MS patients. This study suggested an involvement of the basal ganglia and motor cortex in the pathophysiology of MS and the precuneus is involved in the development of MS (37). Positron emission tomographic scans have shown decreased blood flow to the sensorimotor area in response to lower face vibrations implies abnormal sensorimotor processing in MS patients (14).

Deoxyglucose metabolism in the striatum and thalamus was shown to be increased in MS by positron emission tomography, which was proposed to be related to hyperactivity in the striatum and hypothalamus (38, 39). MS is thought to develop as a result of damage to the brain base, causing an imbalance in dopamine receptor sensitivity (40). MS was also found to be associated with decreased inhibition in the cerebral cortex caused by environmental factors and genetic susceptibility (9, 14, 40). Animal studies have demonstrated that abnormal interactions among trigeminal blink circuits, basal ganglia, and the cerebellum contribute to the disease (41). Although there is no definitive evidence for the existence of MS susceptibility genes, some studies indicate that it is a low penetrance autosomal dominant disorder (33, 42–45).

Silent functional magnetic resonance imaging (MRI) has shown decreased activation of the primary motor cortex (Brodmann Area 4) and premotor cortex (Brodmann Area 6) in the mouth representing areas in MS patients having isolated BSP (14). It might be caused by abnormal control of cranial nerve nuclei in the brain stem by basal ganglia. Resting-state functional magnetic resonance imaging (MRI) has shown altered functional connectivity at rest in widespread brain regions including basal ganglia, cerebellar, primary/secondary sensorimotor, and visual areas in BSP and MS patients. This may reflect a predisposition for defective movement inhibition and sensorimotor integration (46). Transcranial magnetic stimulation electromyographic (EMG) responses study showed that MS had shorter SPs than BSP alone. The shortened SP in facial muscles reflects hypoexcitability of cortical inhibitory neurons in MS (47). The analysis of the semiautomatic rhythmic movements required for chewing and swallowing could reveal the action-related dystonic features. MS patients had an excess duration of muscle activity, frequent cocontraction, loss of rhythmicity during chewing, and abnormalities in the chewing to swallowing transition phase. These abnormalities, similar in type to those encountered in other forms of focal dystonia, maybe the expression of abnormal motor control of basal ganglia over mastication-related movement pattern generators of the brainstem (48).

The roles of the basal ganglia and thalamus are essential for coordination of eyelid movements, and the blink reflex, as well as the cerebello-thalamo-cortical pathways and cortico-striato-pallido-thalamo-cortical pathways, as demonstrated on functional MRI. Blink reflex and masseter inhibitory reflex have also been studied in BSP and MS patients (47, 49–53). Reflexive blinking in BPS is associated with increased activation in the caudate nucleus and sensorimotor cortices and the association between decreasing neural response during reflexive blinking in the cerebellum and disease duration suggests a loss of inhibition within the sensorimotor corticobasal ganglia network (51). Studies found that BSP is associated with a lesion of a complex neural network-cortex-thalamus-globus pallidus-cortex-and does not correspond to a single, unique lesion. This network is connected with ascending and descending sensory-motor pathways and motor nuclei (54). The blink reflex consists of an early, pontine R1-component and a late, medullary R2-component (55). Studies found that the latency of the early (R1) and late (R2) components of the blink reflex and the corneal reflex was normal in MS patients. However, the amplitude and the duration of the R1 and R2 and the duration of the corneal reflex were increased (56, 57). This has been demonstrated in BSP by coupling a train of electric shocks to the supraorbital nerve during the R2 (the second and major response) of the blink reflex (58). This causes an increase in the R2 amplitude that is enhanced in BSP patients relative to control subjects (4, 59). The studies about the blink reflex circuit in MS and BSP supported that the loss of inhibition within the blink reflex circuit contributed to the pathophysiology of these diseases.

The pathogenesis and the pathophysiology of OMD are not well-known (60). The study compared the “movement-related cortical potentials” (MRCPs) between 6 OMD patients and 8 normal subjects and found that MRCP amplitudes over central and parietal areas for mouth opening and lateral movements were significantly reduced compared to normal subjects, which implies that impaired cortical preparatory process for jaw movements exist in OMD (61). Although scientists attempted to elucidate the pathophysiology of OMD using several neuroimaging techniques, its etiology remains unclear (61, 62). Further research is needed to explore the pathophysiology of OMD to develop better treatments.

## Genetics

To date there have been no specific pathogenic genes identified for MS, BSP, and OMD; however, given the close relationship between MS, BSP, OMD, and dystonia, dystonia susceptibility genes may play an important role in disease development.

To summarize the mutations related to MS, BSP, and OMD, we searched the PubMed database using the following search strategies: (Meige syndrome^*^[text word] OR blepharospasm [text word] OR oromandibular dystonia [text word] OR “Meige syndrome” [MESH] OR “blepharospasm” [MESH] OR “oromandibular dystonia” [MESH]) AND (mutation^*^ [text word] OR polymorphism^*^[text word] OR variant^*^[text word]). Candidate genes that have been previously linked to MS, BSP, and OMD are shown in Table 1 (42, 45, 63, 64, 68, 86).

**Table 1:** The susceptibility genes of MS, BSP, OMD.

**Gene**	**Phenotype**	**Ethnic**	**Study type**	**Mutation**	**Sample size (mutation/total patients)**	**References**	**Findings and significance**
*THAP1*	MS	Chinese	Cohort study	c.489C>G, p.L63L	3/44	Song et al. (63)	A silent change [c.489C>G (p.L63L)] in exon 3 was identified in three MS patients and further studies are needed to confirm.
	MS	Greek	Case-control	c.208 A>G, p.K70E	1/70	Xiromerisiou et al. (64)	This mutation was not seen in Greek controls and has not been reported in any control series published or available on-line.
	MS	American	Case report	c.377_378delCT, p.Pro126Args*2	1/1	Park et al. (65)	Whole-exome sequencing revealed a frameshift mutation, confirmed as a novel 2–base pair deletion mutation in exon 3 of the THAP1 gene (heterozygous exon 3 c.377_378delCT, p.Pro126Args*2) via full sequencing analysis of DYT6.
	BSP	German	Cohort study	c.-237_236delinsTT	2/92	Lohmann et al. (66)	The non-coding variants c.-237_236delinsTT, we confirm that there is no significant association with BSP
	BSP	American	Case-control	c.71+9C>A, rs200209986	2/198	Vemula et al. (67)	*In silico* and minigene analyses indicated that c.71+9C>A alters THAP1 splicing. This variant is a risk factor.
	BSP	Greek	Case-control	c.-40T>C	1/70	Xiromerisiou et al. (64)	This mutation is possible to be implicated in the regulation of gene expression.
	BSP	American	Case-control	c.-42C>T	1/70	Xiao et al. (68)	This 5′UTR variant could also exerts effects on splicing fidelity or expression levels.
	BSP	Indian	Case-control	c.*157 T > C (c.-40 T>C)	1/10	Giri et al. (69)	Potential effect of this c. *157 T > C nucleotide alteration on THAP1 mRNA stability or the binding site alteration for regulatory proteins and miRNA.
	BSP	Chinese	Case-control	c.224A>T, p.N75I; c.449A>C, p.H150F	2/102	Cheng et al. (70)	These two variants did not change RNA expression, further functional study is needed.
	OMD	Serbian	Cohort study	c.109_132dup, p.E37_N44dup; c.62C>G, p.S21C	2/36	Dobričić et al. (71)	The duplicated residues are involved in forming of a loop (L2: Phe25-Lys32)-helix (H1: Cys33-Val40)-loop (L3: Arg41- Ser51) structure. *In silico* analysis using software Mutation Taster predicted p.Ser21Cys mutation to be pathogenic. This mutation within the THAP domain abolishes THAP1/TOR1A inter- actions *in vivo*.
	OMD	American	Case-control	c.71+9C>A	1/18	Xiao et al. (68)	Intron 1 (c.71+9C>A) variant could also exerts effects on splicing fidelity or expression levels.
	OMD	Chinese	Case-control	c.267G>A, p.K89K/F25fs53X	1/102	Cheng et al. (70)	Semi-quantitative real-time PCR indicated that a novel silent mutation (c.267G>A) decreased the expression of THAP1 in human lymphocytes.
	OMD	American	Case-control	c.71+9C>A, rs200209986	1/18	Vemula et al. (67)	*In silico* and minigene analyses indicated that c.71+9C>A alters THAP1 splicing. This variant is a risk factor.
*TOR1A*	BSP	Italian and American	Case-control	rs2296793; rs1182; MtDEL	89/190;80/196;62/123	Clarimon et al. (42)	No relation was found between these variants and BSP
	BSP	American	Case-control	ΔGAG	0/67	Xiao et al. (72)	No mutation was found in this study.
	BSP	Italy	Cohort study	191G/T, rs1182	140/401	Defazio et al. (73)	There was significant association between this variant and BSP.
	OMD	American	Case-control	ΔGAG	0/16	Xiao et al. (72)	No mutation was found in this study.
	OMD	American	Case report	c.613T>A, p.F205I	1/1	Calakos et al. (74)	This mutant TOR1A was functionally impaired was obtained using cell culture expression studies.
*GNAL*	MS	German	Cohort study	p.Gly213Ser	1/318	Kumar et al. (75)	This variant predicted to be pathogenic *in silico*, were absent in ethnically matched control individuals, and impaired Gαolf coupling to D1 receptors in a bioluminescence energy transfer (BRET) assay.
	OMD	Chinese	Cohort study	c.-41T>C	1/13	Ma et al. (76)	One patient has this variant which presents in healthy controls.
	OMD	Italian	Cohort study	c.628G>A, p.Asp210Asn	2/2	Carecchio et al. (77)	*In silico* prediction programmes as well as segregation analysis confirmed its pathogenicity.
*CACNA1A*	BSP	United States, Poland, and Italy	Cohort study	c.7261_7262delinsGT	4/31	Tian et al. (78)	The identified amino acid substitution is located in the C-terminal, intracellular domain of the encoded voltage-dependent P/Q-type calcium channel subunit α-1A, which is conserved among mammals.
*REEP4*	BSP	United States, Poland, and Italy	Cohort study	c.109C>T	4/31	Tian et al. (78)	This variant alters an amino acid that is highly conserved among vertebrates as shown by the multiple pairwise alignments generated with Clustal Omega.
*TOR2A*	BSP	United States, Poland, and Italy	Cohort study	c.568C>T, p.Arg190Cys	4/31	Tian et al. (78)	This variant alters an amino acid that is highly conserved among vertebrates as shown by the multiple pairwise alignments generated with Clustal Omega.
*ATP2A3*	BSP	United States, Poland, and Italy	Cohort study	c.1966C>T	4/31	Tian et al. (78)	Predicted to be highly deleterious by all *in silico* analysis.
*HS1BP3*	BSP	United States, Poland, and Italy	Cohort study	c.94C>A	2/31	Tian et al. (78)	Predicted to be highly deleterious by all *in silico* analysis.
*GNA14*	BSP	United States, Poland, and Italy	Cohort study	c.989_990del	2/31	Tian et al. (78)	This GNA14 variant is predicted to be deleterious.
*DNAH17, TRPV4, CAPN11, VPS13C, UNC13B, SPTBN4, MYOD1*, and *MRPL15*	BSP	United States, Poland, and Italy	Cohort study	–	>2/31	Tian et al. (78)	Variants found in patients but lack functional studies to confirm.
*ANO3*	BSP	American	Case report	c.702C > G	1/1	Blackburn et al. (79)	Predict this missense mutation to be deleterious, possibly damaging, and disease causing.
*BDNF*	BSP	Spanish	Case-control	Val66Met	106/252	Gómez-Garre et al. (80)	No relation was found between these variants and BSP.
	BSP	Chinese	Case-control	Val66Met	32/37	Chen et al. (81)	Significant differences were found in the genotype and minor allele frequencies of Val66Met SNP between BSP patients and controls.
*ATXN8*	OMD	American	Case report	CTG•CAG	1/1	Ushe et al. (82)	Mutation of chromosome 13q21 with a CTG•CAG expansion that is transcribed in both directions causing mutations in both ATXN8 and ATXN8OS (coding for Ataxin-8 and a noncoding sequence respectively)
*DRD5*	BSP	Britsh	Case-control	Allele 2 of a DRD5 dinucleotide repeat	10/88	Misbahuddin et al. (45)	Allele 2 of a DRD5 dinucleotide repeat was significantly associated with BSP.
	BSP	Italian and American	Case-control	Allele 2 of a DRD5 dinucleotide repeat	-	Clarimon et al. (42)	No relation was found between this variant and BSP.
*CYP1A2*	BSP	Greek	Case-control	rs762551	110/206	Siokas et al. (83)	CYP1A2 rs762551 is associated with BSP
*ADORA2A*	BSP	Greek	Case-control	rs5760423	129/206	Siokas et al. (83)	No significant differences in allele and genotype frequencies regarding ADORA2A rs5760423 be- tween the patients with BSP and controls were found.
*SYNE1*	BSP	Chinese	Cohort	p.Gln6893Lys, p.His1813Arg, p.Pro3990Ala, p.Glu3457Lys, p.Pro5813Arg, p.Arg8000His, p.Asp4358Asn	7/20	Dong et al. (84)	SYNE1 gene mutations in seven cases, and need further investigation.
*CIZ1*	BSP	Chinese	Cohort	c.2380C>T(p.Arg794Cys), c.400C>T(p.Pro134Ser)	2/20	Dong et al. (84)	CIZ1 gene mutations in two cases, and need further investigation.
*ARSG*	BSP	Greek	Case-control	rs11655081	32/201	Siokas et al. (85)	Lack of association of the rs11655081 ARSG gene with BSP.

*MS, Meige syndrome; BSP, Blepharospasm, OMD,oromandibular dystonia*.

Mutations in *torsion dystonia* (*DYT*)*1* (*TOR1A* encoding torsion (Tor)A, Online Mendelian Inheritance in Man [OMIM] ID: 605204) and *DYT6* (*THAP1* encoding THAP domain-containing 1, OMIM ID: 609520) often affect the craniocervical muscle and may contribute to the pathophysiology of MS (87–89). Evidence from gene expression studies in rodents and brain functional imaging suggests that DYT1 dystonia is a disorder of neural network development. At the cellular level, TorA mediates the interaction between the nuclear envelope and cytoskeleton, and mutations in TorA can indirectly prevent transcription factor entry into or exit from the nucleus (50, 90). TOR1A was shown to be critical for synapse formation and hence, for organizing connectivity in the spinal sensorimotor circuit (91); *TOR1A* mutations are observed in cases of early-onset torsion dystonia (92) and have been linked to late-onset focal, segmental, and multifocal dystonia including BSP, OMD, and MS (42, 72, 73). TOR1A mutations were investigated in BSP and OMD patients in four publications (42, 72–74). ΔGAG of TOR1A mutation was not identified in 67 BSP and 16 OMD patients, which implies that this mutation may not be associated with BSP and OMD (72). A case report found c.613T>A (p.F205I) mutation in OMD and this mutation produces frequent inclusions when expressed in cultured cells, a phenotype shared by the Δ E mutant TOR1A, but not wildtype protein (68). A case-control study found a significant association between rs1182 of *TOR1A* and BSP (62). While another study suggested that rs2296793, rs1182, and MtDEL of *TOR1A* were not associated with BSP (42). Up to now, limited evidence showed the role of *TOR1A* mutation in MS, BSP, and OMD.

THAP1 is a transcription factor expressed in the central nervous system; mutations in this protein lead to aberrant eukaryotic initiation factor 2α (eIF2α) signaling, mitochondrial dysfunction, defects in neuronal projection and axon guidance, and long-term synaptic depression, which are common features of various forms of primary dystonia (93, 94). THAP1 regulates cell proliferation and the G1/S checkpoint by modulating retinoblastoma/E2F target genes (95, 96), and THAP1 overexpression and knockdown in endothelial cells inhibit cell proliferation (95). Some mutations commonly found in BSP, OMD, and MS are shown in Table 1 (63, 64, 66, 68, 70). Now *THAP1* was the most studied gene for the diseases and was the most likely susceptibility gene of these diseases. A silent change [c.489C>G (p.L63L)] in exon 3 was identified in three MS patients and further studies are needed to confirm its effects on gene function (53). A case-control study found the c.208 A>G (p.K70E) mutation in one of 70 MS patients, and this mutation was not seen in Greek controls and has not been reported in any control series published or available on-line (52). Whole-exome sequencing revealed a frameshift mutation in one MS patient, confirmed as a novel 2–base pair deletion mutation in exon 3 of the THAP1 gene (heterozygous exon 3 c.377_378delCT, p.Pro126Args^*^2) via full sequencing analysis of DYT6 (65). *In silico* and minigene analyses indicated that c.71+9C>A alters *THAP1* splicing and this variant is a risk factor (67). c.-40T>C was identified in two BSP patients in two publications, the potential effect of this nucleotide alteration on *THAP1* mRNA stability or the binding site alteration for regulatory proteins and miRNA (64, 69). c.-42C>T of *THAP1* was found in one BSP patient, which is a 5′UTR variant which could also exert effects on splicing fidelity or expression levels (68). p.N75I and p.H150F mutations were identified in two BSP patients and these two variants did not change RNA expression, the further functional study is needed (70). Moreover, a series of case-control studies in OMD patients also identified some mutations, although the frequencies are rare. The mutation c.109_132dup(p.E37_N44dup) duplicated residues that involved in the forming of a loop (L2: Phe25-Lys32)-helix (H1: Cys33-Val40)-loop (L3: Arg41- Ser51) structure (71). Semi-quantitative real-time PCR indicated that a novel silent mutation (c.267G>A) decreased the expression of THAP1 in human lymphocytes (70). *In silico* and minigene analyses indicated that c.71+9C>A alters THAP1 splicing or expression levels in two publications (67, 68). Based on the current study, we conclude that this gene mutation is rare in patients, and more large-scale case-control studies are needed to search for the mutation and the related functional studies of the mutation.

*GNAL* encodes guanine nucleotide-binding protein G(olf) subunit α, which mediates odorant signaling in the olfactory epithelium; it is located on chromosome 18p centromeric to the *DYT7* locus of focal dystonia (97, 98). *GNAL* mutations have been linked to abnormalities in dopamine type 1 and/or adenosine A2A receptor transmission that is thought to result in dystonia (98, 99), and have been detected in patients with OMD and MS (76, 77, 100). Kumar et al. carried out a cohort study and found p.Gly213Ser mutation in one MS patient. This variant was predicted to be pathogenic *in silico*, was absent in ethnically matched control individuals, and impaired Gαolf coupling to D1 receptors in a bioluminescence energy transfer (BRET) assay (75). One patient has c.-41T>C mutation which also presents in healthy controls (76). *In silico* prediction programmers as well as segregation analysis confirmed the pathogenicity of p.Asp210Asn mutation in two OMD patients (77).

Brain-derived neurotrophic factor (BDNF) is a member of the nerve growth factor family (101) that is necessary for the survival of striatal neurons and for synaptic plasticity in the adult brain (101). The *BDNF* Val66Met mutation is associated with an increased risk of BSP development and may protect against BSP (81), although other studies have found no association between the *BDNF* Val66Met variant and BSP (80, 102).

*ANO3* encodes anoctamin-3, which belongs to the anoctamin family of Ca^2+^-activated chloride channels and is highly expressed in the striatum, hippocampus, and cortex. ANO3 modulates neuronal excitability (79, 103), and c.702C > G mutation has been observed in one BSP patient (79). Functional predicting showed this missense mutation be deleterious, possibly damaging, and disease-causing (79). A recent case-control study showed the rs11655081 of *ARSG* was not associated with BSP (104). *SYNE1* gene mutations in seven BSP, CIZ1 gene mutations in two BSP patients were identified and the role of these mutations in the etiology of BSP needs further investigation (84, 85).

A case reported a mutation in *ATXN8* in a BSP patient (82). This mutation of chromosome 13q21 with a CTG•CAG expansion that is transcribed in both directions causing mutations in both ATXN8 and ATXN8OS (coding for Ataxin-8 and a noncoding sequence, respectively) (82). Whole-exome sequencing of 31 subjects with BSP from 21 independent pedigrees identified mutations in several genes including *CACNA1A* encoding calcium voltage-gated channel subunit α1 A (NM_001127222.1: c.7261_7262delinsGT, p.Pro2421Val), *REEP4* encoding receptor accessory protein 4 (NM_025232.3: c.109C>T, p.Arg37Trp), *TOR2A* (NM_130459.3: c.568C>T, p.Arg190Cys), and *ATP2A3* encoding ATPase sarcoplasmic/endoplasmic reticulum Ca^2+^ transporting 3 (NM_005173.3: c.1966C>T, p.Arg656Cys). Deleterious mutations in *HS1BP3* encoding hematopoietic cell-specific Lyn substrate 1-binding protein 3 (NM_022460.3: c.94C>A, p.Gly32Cys) and *GNA14* encoding G protein subunit α14 (NM_004297.3: c.989_990del, p.Thr330ArgfsTer67) were present in a father and son with segmental CCD that initially presented as BSP. Additionally, deleterious variants of genes encoding dynein axonemal heavy chain (*DNAH17*); transient receptor potential cation channel subfamily V member 4 (*TRPV4*); calpain 11 (*CAPN11*); vacuolar protein sorting 13 homolog C (*VPS13C*); unc-13 homolog B (*UNC13B*); spectrin β, non-erythrocytic 4 (*SPTBN4*); myogenic differentiation 1 (*MYOD1*); and mitochondrial ribosomal protein L15 (*MRPL15*) have been found in two or more unrelated BSP patients (78). Despite the identification of many gene mutations in BSP, OMD, and MS patients, the mutation rates of these genes are low and it is a lack of functional experiments of mutant genes. More large-scale whole-exome sequencing studies are needed to search for the mutation and the related functional studies of the pathogenic mutations also need to confirm their roles in these diseases.

## Clinical Management

There is no curative treatment for MS, BSP, or OMD. Current treatments include botulinum neurotoxin (BoNT) therapy, oral medication, and surgical intervention.

### BoNT Therapy

The most common drug treatment for MS, BSP, and OMD is the injection of BoNT. BoNT-A was first used for the treatment of idiopathic BSP and is currently the preferred therapeutic approach owing to its high efficacy and few side effects (105). BoNT-A has been approved by the U.S. Food and Drug Administration for the treatment of BSP and CD while BoNT-B has been approved for CD (106). The first-time BoNT injections should use minimum effective starting doses, which helps to prevent side effects such as excessive focal muscle weakness (107). The starting doses for each toxin were the same (1.25–2.5 U per site), but the maximum doses used within MS, BSP, and OMD are variable. In common practice, minimal side effects are seen when starting at 5 U per site (107). Some large safety studies have shown efficacy and no significant long-term side effects. Recent guidelines summarized the levels of evidence for BSP and OMD (106). OnaBoNT/A and incoBoNT/A were approved by the U.S. Federal Drug Administration (FDA) for BSP treatment. OnaBoNT/A and aboBoNT/A were approved by FDA for OMD treatment (107). Recent long-term safety and efficacy in daily clinical practice study found that the treatment of BSP and MS with onaBoNT/A and aboBoNT/A is safe and effective, also over a long observation period of up to 29 years (27).

The most common side effect is excessive focal muscle weakness, including ptosis and lagophthalmos in BSP patients while eye dryness and diplopia are less common adverse events (107). The most common adverse events in treating OMD are chewing weakness, dysphagia, dysarthria (tongue injections), and dry mouth (diffusion into salivary glands), while generalized weakness, allergic reactions, and flu-like symptoms are rare side effects (107). The main point for the excessive use of BoNT injection is therapeutic resistance that occurs due to antibody production after recurrent and long-term use (14).

### Oral Medications

While BoNT is first-line therapy, clinicians should consider using oral medications as primary therapy when relative contraindications exist, although there have been few multi-center, placebo-controlled, double-blind studies evaluating their clinical utility in MS, BSP, and OMD. Evidence-based reviews have been published (14, 40, 63), but none of the available agents has been tested in rigorously controlled clinical trials. Oral medications that have been used to treat MS, BSP, and OMD include the anticholinergics (e.g., trihexyphenidyl and benztropine), benzodiazepines (e.g., clonazepam, diazepam, and lorazepam), GABA receptor agonist (e.g., baclofen), dopamine precursor such as levodopa, dopamine receptor agonist (e.g., bromocriptine, and tiapride), vesicular monoamine transporter 2 inhibitor (e.g., tetrabenazine), anticonvulsant such as levetiracetam. Eszopiclone and nitrazepam could alleviate the BSP via reacting at those specific subunits (omega-1 and omega-2) of the GABA receptor complex (108). Some case reports found that zolpidem is effective in such patients as it is highly specific for a GABA omega-1 receptor (109, 110). However, some studies found limited efficacy of oral medications such as zolpidem, levetiracetam, and valproate (111, 112). A randomized controlled trial concluded that Levetiracetam does not appear to be efficacious in patients with OMD or cranial dystonia (111). A single-center, double-blind cross-sectional study revealed that valproate had low efficacy in the treatment of MS (112). Zolpidem and Levetiracetam were slightly effective in patients with MS (109, 113, 114). Moreover, the magnitude of improvement typically obtained with commonly used drugs is often modest, such as the anticholinergics (e.g., trihexyphenidyl and benztropine), benzodiazepines (e.g., clonazepam and lorazepam), baclofen, and tetrabenazine (5). Compare to BoNT, oral medication efficacy is at best modest and does not show the same level. Oral medication therapies are further limited by systemic side effects which are not usually seen with BoNT therapy (107). For oral medication in OMD, the recent system review pointed out that no definitive conclusions can be drawn about the type of patients that may benefit, nor about the preferred type or mode of the appliance (115).

### Surgical Treatment

Surgical treatment is an option for patients who are unresponsive to the conventional drugs used to treat MS, BSP, and OMD. Partial resection of the periorbital muscle resulted in long-term improvement; however, this is not the favored therapeutic strategy owing to postoperative complications such as inflammation, aesthetic issues, hematoma, and exposure keratitis among others and the efficacy of BoNT (116–118). Deep brain stimulation (DBS) has gained increasing attention in recent years as a treatment option for intractable dystonia including MS and DYT1 dystonia (18, 119–122). Some studies have demonstrated that DBS is ineffective in a subset of patients with craniocervical segmental dystonia (123–126), but it was found to improve symptoms of MS (104, 127–129). DBS of the globus pallidus internal and subthalamic nucleus was effective in patients with medically refractory MS, including those exhibiting severe preoperative symptoms (130). A recent meta-analysis showed that DBS of the globus pallidus internal and subthalamic nucleus may be an effective therapy for even refractory MS. Higher preoperative scores probably indicate larger improvement and stimulation targets or other clinical factors do not constitute the outcome predictive factors (131). Reported cases of MS treated with DBS are summarized in Table 2; however, given their limited number, more in-depth studies are needed to validate the clinical utility of this method. BoNT is still the main treatment option for BSP. When it fails, there are not many options. Given the experience of BSP improving with DBS of the globus pallidus internal and subthalamic nucleus in patients with MS, DBS surgery can be an acceptably effective therapy for patients with isolated BSP. The risk of the procedure should be weighed cautiously against the potential benefit.

**Table 2:** Case series of MS, BSP, and OMD patients undergoing DBS.

**References**	**Type of patient**	**Number of patients**	**Site of stimulation**	**Therapeutic effect**
Muta et al. (132)	MS	1	GPi	Improvement while remaining refractory to pharmacotherapy and bilateral thalamotomy
Foote et al. (126)	MS	1	Gpi	Improvement at 6-month follow up
Houser and Waltz (125)	MS	1	Gpi	Substantial improvement
Ostrem et al. (123)	MS	6	Gpi	Improvement of dystonia and slight worsening of motor function was reported in previously non-dystonic body regions in four patients
Hebb et al. (124)	MS	1	Gpi	Sustained relief of dystonia 1 year after cessation of DBS
Blomstedt et al. (121)	MS	1	Gpi	No improvement in axial symptoms but blepharospasm was abolished
Loher et al. (133)	MS	1	Gpi	Long-term symptomatic and functional improvement
Sensi et al. (134)	MS	9	Gpi	Significant improvement at 6 months and better outcome
Woehrle et al. (135)	MS	1	Gpi	Improvement
Inoue et al. (116)	MS	1	Gpi	Sustained long-term improvement (N80%) for 10 years
Ghang et al. (136)	MS	11	Gpi	Effective for intractable MS without significant side effects
Lyons et al. (137)	MS	4	Gpi	Effective for medically refractory MS
Markaki et al. (18)	MS	1	Gpi	Improvement by 70% in movement score and 93.33% in disability score
Romito et al. (138)	MS	1	Gpi	Progressive and sustained improvement of dystonia at 38-month follow-up
Sako et al. (119)	MS	5	Gpi	Significant improvement in movement and disability scales
Reese et al. (120)	MS	12	Gpi	Good effect persisting for up to 6 years
Tai et al. (139)	MS	1	Gpi	Good effect persisting for 36 months
Limotai et al. (140)	MS	6	Gpi	Low-frequency stimulation (100 Hz) was effective in two patients, with two patients experiencing a 20% benefit
Sobstyl et al. (141)	MS	3	Gpi	Burke-Fahn—Marsden dystonia rating scale total disability score was reduced by 34% and 47% at short- and long-term follow-ups, respectively
Bae et al. (142)	MS	1	Gpi	Excellent improvement in speech with no adverse events
Wang et al. (130)	MS	4	Gpi or STN	Significant improvement
Zhao et al. (143)	OMD	1	STN GPi	STN-DBS seemed to induce dyskinesia, which made the patient felt uncomfortable although stimulation was slight. On the contrary, GPi-DBS stimulation relieved her discomfort.
Yamada et al. (144)	BSP	9	GPi	15 months after the operation, his preoperative scores on the Burke-Fahn-Marsden Dystonia Rating Scale (=8 points) decreased to 1 (87.5% improvement). The present study demonstrates the applicability of GPi-DBS for treating blepharospasm presenting as focal dystonia.
Santos et al. (145)	BSP	1	GPi	Blepharospasm improved
Sobstyl et al. (104)	MS	6	Gpi	Significant improvement
Luthra et al. (146)	BSP	1	Gp	This case illustrates successful treatment of blepharospasm with pallidal stimulation.
Zhan et al. (147)	MS	15	STN	Immediate improvement in symptoms after stimulation; four adverse events recorded in three patients, all of which were resolved without permanent sequelae
Horisawa et al. (129)	MS	16	Gpi	Significant improvement
Aires et al. (128)	MS	2	Gpi	Dystonia was improved by 68% in Patient 1 and by 96% in Patient 2, whereas disability was improved by 77%−92% at 24-month follow-up
Yao et al. (127)	MS	15	STN	MS patients (*n* = 14) showed improved BFMDRS score
Shu et al. (148)	MS	1	Gpi	Significant improvement in symptoms
Wang et al. (149)	MS	20	Gp or STN	Good outcome in nine patients and poor outcome in 11 patients
Tian et al. (150)	MS	17	Gpi or STN	Both the STN and Gpi could be effective targets of DBS for MS.
Hao et al. (151)	MS	22	GPi	Bilateral pallidal neurostimulation is a beneficial therapeutic option for refractory MS, which could improve the motor symptoms except for depression and sleep quality.
Ouyang et al. (152)	MS	15	STN	STN-DBS was not only able to improve patients' motor symptoms, but also their sleep status.

*DBS, deep brain stimulation; GPi, globus pallidus internal; STN, subthalamic nucleus; MS, Meige syndrome; BSP, Blepharospasm; OMD, oromandibular dystonia*.

## Conclusions

MS is complex dystonia that includes BSP and OMD. Due to its low incidence, possible genetic heterogeneity, and late age of onset, it is difficult to obtain complete case data in families and, consequently, to identify genetic markers and susceptibility genes. Indeed, no causative genes have been confirmed to date. The viable treatment option that is currently available for MS is repeated injections of BoNT. DBS is an option for patients who are unresponsive to conventional drugs. The application of high-throughput, genome-wide analytical approaches is expected to reveal novel disease markers and potential therapeutic targets, thereby providing a basis for the development of more effective drugs that can bring clinical relief of symptoms and improve the quality of life of BSP, OMD, and MS patients.

## Author Contributions

JQ and QQ: conceptualization. HM, YS, and LY: collection and analysis of bibliography. JQ, QQ, HM, YS, and LY: writing original draft. All authors have read and agreed to the published version of the manuscript.

## Conflict of Interest

The authors declare that the research was conducted in the absence of any commercial or financial relationships that could be construed as a potential conflict of interest.
